# Interaction between polymorphisms in *MBP* and *EIF2B5* genes is significantly associated with Alzheimer’s disease

**DOI:** 10.1002/alz.093410

**Published:** 2025-01-03

**Authors:** Anatoliy I Yashin, Deqing Wu, Konstantin Arbeev, Hongzhe Duan, Rachel Holmes, Olivia Bagley, Svetlana Ukraintseva

**Affiliations:** ^1^ Social Science Research Institute, Duke University, Durham, NC USA

## Abstract

**Background:**

Results of recent analyses indicate that axon demyelination may play an important role in AD pathology. The *MBP* gene encodes a myelin basic protein involved in axon myelination in the nervous system including the central nervous system. Polymorphisms in this gene, as well as variations in expression, have been associated with multiple sclerosis (MS). Evidence of the involvement of this gene in Alzheimer’s disease (AD) is emerging. The *EIF2B5* gene codes for a subunit of eukaryotic translation initiation factor 2B (eIF2B), a master regulator of translation. Recent studies connected *EIF2B5* and its polymorphisms with various health disorders including MS and AD, as well as hypomyelination. The latter was also associated with reduced MBP. Considering this, and that both *MBP* and *EIF2B5* have been associated with AD, we hypothesized that the interplay between these two genes might also be associated with AD.

**Methods:**

To test this hypothesis, we analyzed data on genotyped individuals (males and females combined) from the Health and Retirement Study using a logistic regression model with the interaction term. Education, smoking status, sex, and the first five principal components were included as observed covariates. The binary AD trait was defined as “0” if AD onset was observed at any age, and as “1” if a person survived to age 85 and did not get AD during their life. Linkage disequilibrium (LD) test and SNP‐clumping procedures were used to reduce the number of tests in SNPxSNP interaction analysis and make Bonferroni correction less conservative.

**Results:**

We found a statistically significant positive association of the interaction between intronic rs12959006 in *MBP* gene and missense variant rs843358 in the *EIF2B5* gene with AD. The rs12959006 was associated with MS and rs843358 in survival from cancer in the literature. Several other SNPs that are in high LD with the SNPs found in this study have also been separately associated with health traits in the literature.

**Conclusion:**

The results of this study support the hypothesis that the interaction between MBP and EIF2B5 variants is significantly associated with AD. Our finding implies that regulation of translation and axon demyelination jointly contribute to AD.

